# Functional characterization of *Capsicum chinense* vanillin aminotransferase: Detection of vanillylamine-forming activity from vanillin

**DOI:** 10.1016/j.bbrep.2024.101692

**Published:** 2024-03-25

**Authors:** Yasuo Kato, Taiji Nomura

**Affiliations:** Biotechnology Research Center and Department of Biotechnology, Toyama Prefectural University, 5180 Kurokawa, Imizu, Toyama, 939-0398, Japan

**Keywords:** Capsaicin, Biosynthesis, γ-aminobutyric acid, Vanillylamine, Vanillin aminotransferase

## Abstract

In capsaicin biosynthesis, vanillin aminotransferase (VAMT; EC 2.6.1.119) catalyzes the conversion of vanillin (V) to vanillylamine (VA). *In vitro* analysis of the recombinant VAMT enzyme has been reported; however, this enzyme catalyzed only the V-forming reaction and not the VA-forming reaction, which is inconsistent with the postulated pathway for capsaicin biosynthesis. In this study, we expressed, purified, and characterized functional recombinant VAMT of *Capsicum chinense* cv. Habanero from an *Escherichia coli* strain. The enzyme catalyzed reversible transamination between V and VA, and its VA-forming activity was high when γ-aminobutyric acid (GABA) was used as an amino donor. The enzyme exhibited maximum activity at pH 8.0 and 55 °C, and was stable up to 60 °C over a pH range from 4.5 to 8.0. The enzyme was stable in the presence of various chemicals and metal ions. The enzyme accepted several 4–8-carbon long primary amines and ω-amino acids with carbon chains longer than 4 as amino donors despite the narrow specificity of the amino acceptor. Based on its kinetic attributes and localization, VAMT appears to have evolved from GABA-aminotransferase to catalyze reversible transamination between V and VA, and is responsible for VA biosynthesis using GABA as an amino donor in the cytosol of capsicum fruit cells.

## Highlights

• Vanillin aminotransferase (VAMT) was expressed in recombinant *E. coli* strain.
• VAMT catalyzed reversible transamination between vanillin and vanillylamine.
• VAMT might be evolved from GABA-aminotransferase.

## Graphical abstract

**Figure:**
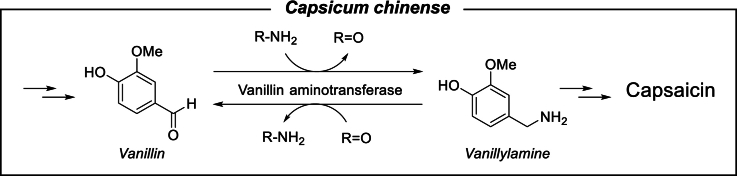

## 1 Introduction

Capsicum belongs to the Solanaceae family, which includes several species in the genus *Capsicum*. Capsicum has a long history of human use: it has been cultivated in Peru and other Latin American countries since 8000–7000 B.C. Capsicum spread throughout the world over a small duration of 100 years, from 1500 to 1600 B.C., and has had the most dramatic impact on all foods and cultures [1]. Five species of *Capsicum annuum* are the species cultivated currently, and these are cultivated worldwide not only because of their adaptability and high productivity, but also because of the pungent taste they offers at low prices. Capsaicin, a hydrophobic alkaloid, was first isolated by Bucholz in 1816 from the *C. annuum* fruit as its pungent component by soaking it in organic solvents [2]. In 1846, Thresh crystallized a pungent stimulant and named it capsaicin [3]. After its chemical structure was determined in 1919 [4], several pungent compounds, including dihydrocapsaicin, nordihydrocapsaicin, homocapsaicin, and homodihydrocapsaicin were isolated from capsicum fruits and are referred to as capsaicinoids [5]. The biological functions of capsaicin have been widely studied because of its heat-producing effects, such as increased body temperature and perspiration [6], antioxidant [7], and antimicrobial [8] effects.

**Fig. 1:**
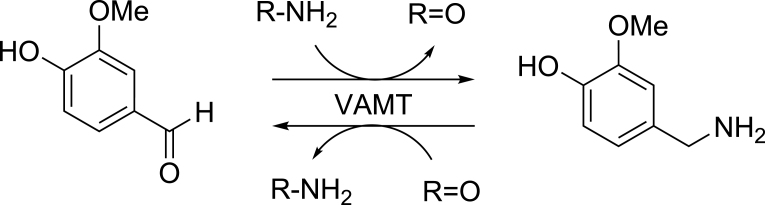
Reaction catalyzed by VAMT.

The biosynthetic pathway of capsaicin was postulated using feeding experiments [9], although it has not yet been fully elucidated. It starts from l-Phe and l-Val, which undergo several enzymatic reactions to form the precursors vanillylamine (VA) and 8-methyl-6-nonenoyl-CoA, respectively, and capsaicin is biosynthesized by their condensation. The enzyme vanillin aminotransferase (VAMT; EC 2.6.1.119, Fig. 1) is involved in the formation of VA from vanillin (V), because the gene (*vamt*) encoding this enzyme isessential for capsaicin biosynthesis [10]. In addition, capsaicin levels in *C*. *annuum* were drastically reduced when *vamt* was deleted [11]. Recently, *in vitro* analysis of recombinant VAMT was reported; however, the enzyme catalyzes only the deamination of VA to V (using pyruvate and oxaloacetate as amino acceptors), and not the VA-forming reaction from V (using L-Ala as an amino donor) [12]. This is inconsistent with the postulated pathway for capsaicin biosynthesis. As capsaicin is biosynthesized from V through VA, it is easy to estimate whether VAMT catalyzed the formation of VA from V. Moreover, the recombinant enzyme did not accept γ-aminobutyric acid (GABA) as an amino donor [12]; nevertheless, VAMT shares 76–83% amino acid identities with known plant GABA aminotransferases from *Arabidopsis thaliana* [13], *Malus domestica* (apple) [14], and *Solanum lycopersicum* (tomato) [15]. Indeed, the production of VA using a crude extract of *C. chinense* cv. Habanero was markedly enhanced when GABA was used as an amino donor in the reaction mixture compared with that in the presence of other amino acids [16].

In this study, we re-examined the enzymatic properties of VAMT by expressing and purifying it from a recombinant *Escherichia coli* strain and assaying it using several amino donors, including GABA. We found that the enzyme catalyzed the transamination reaction between V and GABA to form VA. The biological significance of VAMT in capsicum fruit and its involvement in capsaicin biosynthesis are highlighted in this manuscript.

## 2 Materials and methods

### 2.1 Chemicals

Vanillin glucoside (VG) and vanillylamine glucoside (VAG) were synthesized as described previously [17] and in Supplementary Material (Figs. S1 and S2), respectively. All other chemicals including V, VA, succinate semialdehyde (SSA), and PLP (as monohydrate form), were obtained from commercial sources and used without further purification.

### 2.2 Analytical methods

V- and VA-forming reactions were assayed using reversed-phase HPLC under the following conditions: column, Mightysil RP-18 GP Aqua (5 μm, 4.6 mm × 150 mm, Kanto Chemical, Tokyo, Japan); mobile phase, 5% (v/v) CH_3_CN in 100 mM HClO_4_; flow rate, 1 mL/min; detection, 254 nm; and temperature, 25 °C. For analyzing glucoside-type substrates, 2.5% (v/v) CH_3_CN in 100 mM HClO_4_ was used as the mobile phase. HPLC chromatograms of the compounds were shown in Fig. S3.

The protein concentration was determined using a Protein Assay Kit (Bio-Rad, Hercules, CA, USA) with bovine serum albumin as a standard protein. Sodium dodecyl sulfate-polyacrylamide gel electrophoresis (SDS-PAGE) was performed using a PageRun (AE-6531, ATTO, Tokyo, JAPAN). SDS-PAGE Standard (Low MW, Bio-Rad) was used as a molecular size marker. Proteins resolved in the gel were stained using Coomassie Brilliant Blue (CBB R-250).

### 2.3 Measurement of enzyme activity

The enzyme activity was determined based on the increase in product concentrations in the reaction mixture. One unit of enzyme activity was defined as the amount of enzyme responsible for 1 μmol conversion reaction per min. The standard assay mixture for measuring the V-forming activity contained 50 mM potassium phosphate buffer (KPB) (pH 8.0), 0.1 mM PLP, 100 mM pyruvate (sodium form) as an amino acceptor, 5 mM VA as an amino donor, and the enzyme in a total volume of 100 μL. The VA-forming activity was assayed in the same reaction mixture except for the fact that 5 mM V was used as an amino acceptor and 100 mM GABA or L-Ala was used as an amino donor. The reaction was performed at 30 °C for 30 min and was stopped by addition of 100 μL of 500 mM H_3_PO_4_ and 300 μL of H_2_O:CH_3_CN (a 5:1 mixture). The supernatant obtained after centrifugation (18,000×*g*, 10 min) of the reaction mixture was subjected to HPLC analysis.

The optimum pH and temperature for the enzyme reaction were determined by measuring the enzyme activity for 30 min in 100 mM GTA buffer at pH 3.5–10.0 (every 0.5 pH unit) and at 15–60 °C (every 5 °C) in 100 mM KPB (pH 8.0), respectively. The pH and temperature stabilities of the enzyme were determined by incubating the enzyme for 30 min in 100 mM GTA buffer at pH 3.5–10.0 (every 0.5 pH unit) and for 30 min or 4 h at 15–60 °C (every 5 °C) in 100 mM KPB (pH 8.0), respectively. Aliquots of the enzyme were used to measure the remaining activity. V- or VA-forming activities were measured using VA and pyruvate or GABA and V as the amino donor and acceptor, respectively.

The effects of various compounds on the enzyme activity were examined by measuring the V-forming activity under standard assay conditions in the presence of various concentrations of inhibitors, coenzymes, and metal ions.

The effects of various amino donors and acceptors on the enzyme activity were examined by measuring the activity for V- or VA-formation under the standard assay conditions using combinations of VA (5 mM) and various carbonyl compounds (100 mM) or various amine compounds (100 mM) and V (5 mM) as amino donor and acceptor, respectively.

### 2.4 Construction of recombinant *E. coli* strain

*vamt* cDNA was cloned from green fruits of *Capsicum chinense* cv. Habanero which had been grown in Chiba, Japan (purchased from Pepper Friends Co. Ltd). Total RNA was extracted from freeze-dried fruit samples (50 mg DW) using the Isospin Plant RNA kit (Nippon Gene, Tokyo, Japan), DNase I-treated, and repurified by phenol-chloroform treatment and ethanol precipitation. cDNA was synthesized from the purified RNA using the SuperScript III First-Strand Synthesis SuperMix for RT-PCR Kit (Invitrogen, Waltham, MA, USA) and subsequently used as a template for PCR using primers designed based on the published sequences of *vamt* (GenBank accession no. AF085149) [16,18]. PCR was performed as follows: 2 min at 94 °C, followed by 35 cycles of amplification (10 s at 98 °C, 30 s at 55 °C, and 90 s at 68 °C) in a 50 μL reaction mixture containing 24 ng of cDNA, 0.5 μM each of VAMT-Nde-F (5′-CGCGCGGCAGCCATATGGCCAATATTACTAAT-3′) and VAMT-Nde-R (5′-GTCATGCTAGCCATATTACTGCTTCTGAGACTT-3′) primers, 0.2 mM dNTPs, 1 × reaction buffer, and 5 U of KOD Plus Neo DNA polymerase (Toyobo, Shiga, Japan). The amplified PCR product was confirmed to have 100% identity with the known *vamt* sequence [18] and it was inserted into the NdeI site in the pET28a-vector (Novagen, Madison, WI, USA) using an In-Fusion HD Cloning Kit (Takara Bio, Shiga, Japan) to generate pVAMT/pET. The resulting expression plasmid was used to transform *E. coli* Rosetta2 (DE3) strain (Novagen).

### 2.5 Cultivation of recombinant *E. coli* strain and purification of VAMT

Recombinant *E. coli* cells were grown in 200 mL of Luria Bertani medium containing 25 μg/mL kanamycin and 25 μg/mL chloramphenicol on a rotary shaker (200 rpm) at 37 °C for 6 h. When the optical density at 610 nm of the culture reached 0.6, the culture was cooled on ice and isopropyl β-d-1-thiogalactopyranoside was added at a final concentration of 1 mM. The culture was incubated at 18 °C for 20 h at 200 rpm. The following operations were performed at 4 °C. The cells (29.5 g) were harvested by centrifugation (2500×*g*, 5 min) and resuspended in 150 mL of 50 mM HEPES buffer (pH 7.5) containing 200 mM NaCl, 10 mM 2-mercaptoethanol (2-ME), and 10 μM PLP. The cells were disrupted for 15 min using a Kubota-Shoji 9 kHz sonic oscillator (UD-201, Tokyo, Japan) and centrifuged (5000×*g*, 15 min) to obtain the soluble enzyme fraction. TALON His-Tag Purification Resin (8 mL; Clontech, Palo Alto, CA, USA), equilibrated with the same buffer, was mixed with the enzyme solution under gentle agitation for 40 min. The resin was washed with the same buffer (50 mL × 2) and eluted with 30 mL of the same buffer containing 200 mM imidazole. The eluted His-tagged VAMT was digested overnight with 50 U thrombin (Merck, Darmstadt, Germany) to remove the His-tag. The resulting enzyme solution was brought to 25% ammonium sulfate saturation and placed on a Butyl-Toyopearl column (10 mL, Tosoh, Tokyo, Japan) which had been equilibrated with 100 mM KPB (pH 7.5) containing 25% saturated ammonium sulfate solution, 10 mM 2-ME, and 10 μM PLP. The active fractions that were eluted with a linear gradient of ammonium sulfate (20–10% saturation) in the same buffer were combined, concentrated using Amicon Ultra-4 centrifugal filter units (Merck), dialyzed against the 10 mM buffer, and stored at 4 °C.

### 2.6 Measurement of the molecular mass of recombinant VAMT

The purified enzyme was subjected to gel filtration chromatography using a Superdex 200 Increase column (GE Healthcare, Piscataway, NJ, USA) (buffer: 50 mM KPB (pH 7.5) containing 150 mM NaCl, 10 mM 2-ME, and 10 μM PLP; flow rate: 0.5 mL/min), and the native molecular mass of recombinant VAMT was determined relative to the Gel Filtration Calibration Kit HMW (GE Healthcare) as a marker protein.

### 2.7 Enzyme kinetics

The analysis of the kinetics of the reaction for V (VG)-forming reaction was performed in a reaction mixture (100 μL) containing 50 mM KPB (pH 8.0), 0.1 mM PLP, 100 mM pyruvate, and various concentrations of VA (VAG). For VA (VAG)-forming reaction, the reaction mixture (100 μL) comprised 50 mM KPB (pH 8.0), 0.1 mM PLP, 100 mM L-Ala or GABA, and various concentration of V (VG). Apparent *K*_m_ and *V*_max_ values were calculated based on non-linear regression of the data to the Michaelis–Menten equation using Sigma Plot 12.5 (Systat Software, San Jose, CA, USA) after linear confirmation with the Lineweaver–Burk Plot or Hanes–Woolf Plot.

The substrate-binding mechanism was confirmed based on VA-formation with GABA as an amino donor in the following reaction mixture (100 μL) containing 50 mM KPB (pH 8.0), 0.1 mM PLP, 25–250 mM GABA, and 0.1–2.5 mM V. The Lineweaver–Burk plots of GABA concentration and activity at each V concentration were prepared and used to confirm the substrate-binding mechanism.

## 3 Results and discussion

### 3.1 Heterologous expression of VAMT in *E. coli* and detection of reversible aminotransferase activity of the enzyme

**Fig. 2:**
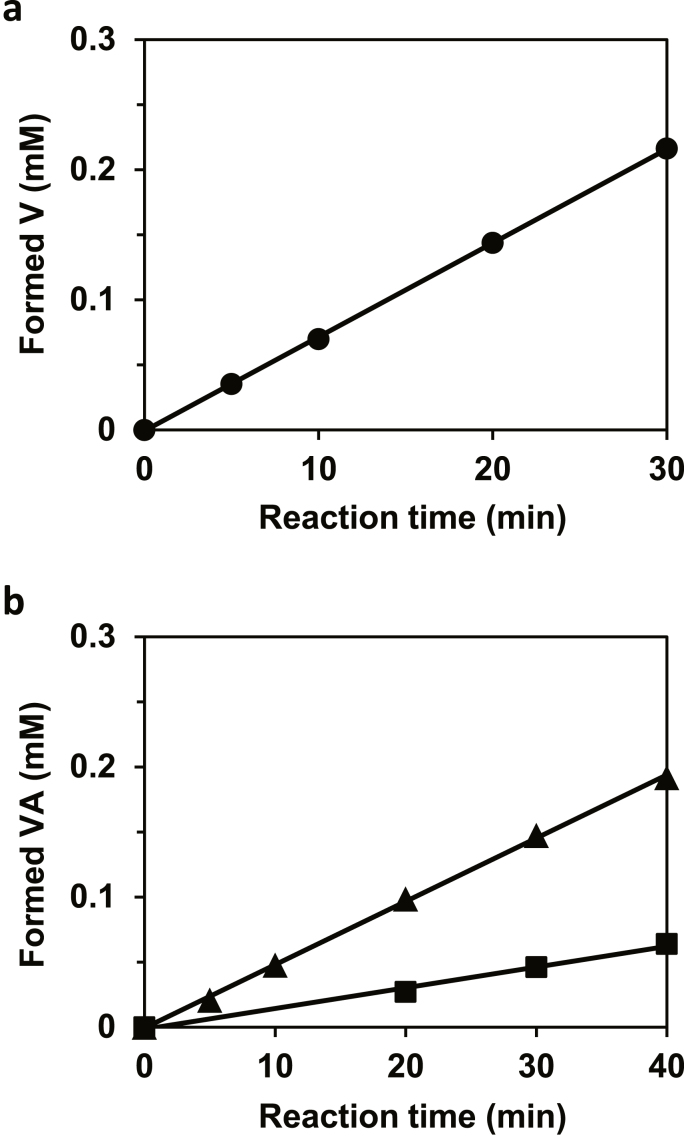
Time course of the reaction catalyzed by VAMT. The reaction mixture (100 μL) was composed of 50 mM KPB (pH 8.0), 0.1 mM PLP, amino acceptor, amino donor, and the crude extract of *E. coli* Rosetta2 (DE3) pVAMT/pET strain containing a) 2.8 μg or b) 2.0 μg of protein. a) V-forming reaction from VA (5 mM) with 100 mM pyruvate as amino acceptor and b) VA-forming reaction from V (5 mM) with 100 mM GABA (▲) or 100 mM L-Ala (■) as amino donors, respectively.

In a previous study [12], recombinant VAMT was prepared using an expression plasmid in which *vamt* was codon-optimized for expression in *E. coli*. Here, we examined the expression of VAMT using *vamt* without codon optimization under the control of a T7-promotor (pVAMT/pET) in *E. coli* Rosetta2 (DE3) strain as a host. Screening of conditions, such as cultivation temperature and time, resulted in efficient expression of the enzyme in its soluble form as assessed using SDS-PAGE. We analyzed the V-forming activity in the soluble fraction by measuring the time course of the formation of V from VA with pyruvate as the amino acceptor. As shown in Fig. 2a, the transamination product V was formed linearly in a time-dependent manner, indicating that the recombinant enzyme exhibited the VAMT activity.

VAMT catalyzes the transamination of V to VA during capsaicin biosynthesis [9]. The VA-forming activity was detected in *Nicotiana tabacum* cells (Solanaceae family) transformed with *vamt* using *Agrobacterium*-mediated transformation [19]. However, Weber et al. [12] reported that recombinant VAMT catalyzes only the V-forming reaction from VA and not the VA-forming reaction from V with several amino donors; nevertheless, the enzyme is an aminotransferase that typically catalyzes the reversible amination-deamination reaction. Here, we re-examined the VA-forming activity in the crude extract using L-Ala and GABA considering the primary structure of VAMT is 80% homologous to GABA aminotransferases from *Arabidopsis*, apple, and tomato [12]. As shown in Fig. 2b, the enzyme reactions proceeded linearly, indicating that VAMT catalyzes the VA-forming reaction from V as well as the V-forming reaction from VA. When GABA was used as an amino donor, the activity was approximately three times higher than that with L-Ala. During the preparation of this manuscript, Nakaniwa et al. reported the functional expression of VAMT in *E. coli* BL21 Star (DE3) using the expression plasmid pET3c and confirmed the detection of the VA-forming activity from V for the recombinant enzyme using GABA as an amino donor [20]. They also used *vamt* with non-optimized codon to express the enzyme. It is unclear why recombinant VAMT expressed using a codon-optimized gene did not catalyze any VA-forming reaction even when GABA was used as an amino donor in the previous study [12].

### 3.2 Purification of VAMT from recombinant *E. coli* strain

**Fig. 3:**
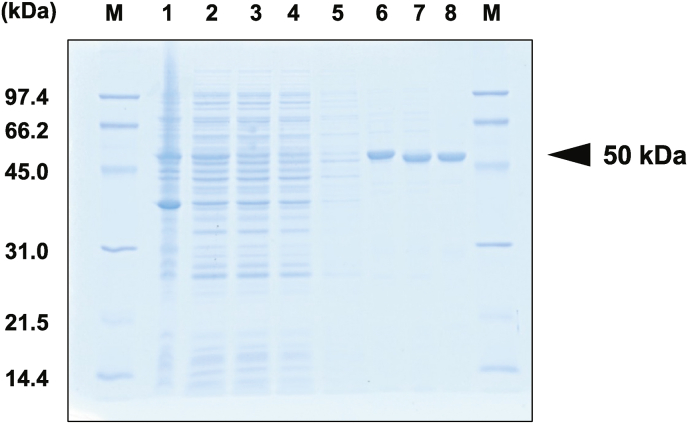
Purification of VAMT from the recombinant *E. coli* Rosetta2 (DE3) pVAMT/pET strain. Proteins from each purification step were separated on a 12.5% SDS-polyacrylamide gel and stained with Coomassie Brilliant Blue (CBB) R-250. Lane M, Molecular size markers; lane 1, insoluble-fraction; lane 2, soluble-fraction (crude extract); lanes 3 and 4, run-through fractions from metal-affinity chromatography; lane 5, wash fraction from metal-affinity chromatography; lane 6, eluate from metal-affinity chromatography; lane 7, protein after thrombin digestion; lane 8, Butyl-Toyopearl fractions.

VAMT was purified from the *E. coli* Rosetta2 (DE3) pVAMT/pET strain grown under optimal culture conditions using TALON affinity and Butyl-Toyopearl column chromatographies (Table 1 and Fig. 3), including thrombin digestion. During purification, 10 mM 2-ME and 10 μM PLP were added to the buffer to stabilize the enzyme. To facilitate purification, the recombinant VAMT was designed to add a peptide (Leu-Val-Pro-Arg-Gly-Ser) containing a His_6_-tag and a thrombin cleavage site effective for tag removal at the *N*-terminal end of the enzyme using an expression vector (pET28a). The His-tag was efficiently removed by thrombin digestion, as evident from SDS-PAGE analysis. The purified samples showed a band of approximately 50 kDa, which is in accordance with the estimated molecular mass of VAMT. The enzyme was purified to homogeneity using cell-free extract from 29.5 g of the recombinant *E. coli* cells in three steps, with a yield of 9.71% and a 57.3-fold purification. The specific activity for the V-forming reaction from VA was 4.38 U/mg. The native molecular mass of VAMT, determined by gel-filtration analysis using a Superdex 200 Increase column, was 92.5 kDa (Fig. S4), which indicates that the enzyme exists as a dimer in its native form because the subunit molecular mass of VAMT is approximately 50 kDa, based on the amino acid sequence and SDS-PAGE analysis (Fig. 3). This result is in agreement with those for GABA aminotransferases from various origins [21].

**Table 1:** Purification of rVAMT.

Step	Total activity (U)	Total protein (mg)	Specific activity (U/mg)*	Yield (%)	Purification (-fold)
Crude	78.1	1020	0.0765	100	1
TALON	37.9	33.2	1.14	48.5	14.9
thrombin	47.1	33.2	1.42	60.3	18.5
Butyl-Toyopearl	7.59	1.73	4.38	9.71	57.3

* V-forming activity from VA using pyruvate as an amino acceptor.

Because the primary structure of VAMT suggests that it is a PLP enzyme, we measured its absorption spectrum. The enzyme exhibited absorption maxima at 280 and 340 nm, with a trace absorption around 400–410 nm which is characteristic of the PLP-holoenzyme, suggesting that the enzyme exists in a PLP-free form (Fig. S5a). Similar absorption profile was observed for the PLP-dependent bacterial ω-amino acid-pyruvate aminotransferase [22]. The addition of GABA to the enzyme solution at a final concentration of 100 mM did not alter the absorption spectrum (Fig. S5b). Absorption spectra were also measured at different pH values (6.0 and 8.0) in KPB, but no differences were observed. This suggests that PLP is not tightly bound to the active center of the enzyme, which differs from the general characteristics of plant GABA-aminotransferases [21].

### 3.3 Enzymatic characterization of VAMT

Figs. S6 and S7 show the pH and temperature for optimal enzyme activity and VAMT stability in V- and VA-forming reactions, respectively. The optimum pH for the enzyme activity was 8.0, and the optimum temperature was 55 °C at pH 8.0. These data were consistent with results obtained for plant aminotransferases [21]. The enzyme was stable over a pH range from 4.5 to 8.0 at room temperature for 30 min and at temperatures up to 50 °C for 4 h; it was stable at 60 °C for 30 min. Based on the results of the optimum temperature, the enzyme appears to be heat-stable and the reaction proceeds without heat inactivation at temperatures between 15 and 40 °C in both the reactions. Based on an Arrhenius plot (Fig. S8) constructed from the optimal temperature graph, we determined the activation energy (*E*_a_) for V-forming reactions to be 46.9 kJ and that for VA-forming reaction to be 51.7 and 60.6 kJ in reactions with GABA and L-Ala as amino donors, respectively. Because the energies for the V- and VA-forming reactions are comparable, there is little difference in the equilibrium between the two reactions.

The enzyme activity for V-forming reaction was measured in the presence of 2 mM of the various chemicals. As shown in Table S1, the enzyme was weakly inhibited by heavy metal ions: after incubation of the enzyme with Cu^2+^ (2 mM), Cu^2+^ (1.25 mM), and Hg^2+^ (2 mM), 5.4%, 23%, and 35% of the original enzyme activity remained, respectively. Inhibition by these ions may be ascribed to their reaction with the sulfhydryl group of the enzyme, although the SH-reagents were inert as inhibitors. Other chelating, serine, and reducing reagents and metal ions did not exert any inhibitory effect. Interestingly, carbonyl reagents that might bind to the aldehyde group of PLP to inactivate the enzyme did not show any inhibitory effect on the reaction, except for NH_2_OH (37% inhibition at 2 mM).

### 3.4 Substrate specificity and kinetic analysis of VAMT

To study the specificity of VAMT for the amino acceptor, we measured the V-forming activity under standard reaction conditions with a series of 100 mM oxo-acids, such as succinate semialdehyde (SSA), glyoxylate, oxaloacetate, and α-ketoglutarate. Pyruvate was found to be the most suitable amino acceptor, whereas SSA, a deaminated form of GABA, showed only 1/33 of the activity, because it is unstable and tends to be spontaneously coverted to cyclic 4-hydroxybutyrolactone, which is inactive as an amino acceptor. The other tested oxo-acids did not act as amino acceptor. Notably, plant GABA aminotransferases with moderate identities to VAMT accept glyoxylate as an amino acceptor, similar to pyruvate [[13], [14], [15]], but VAMT does not.

The specificity of VAMT for the amino donor was examined by employing the standard reaction conditions in VA-forming reaction with a series of 100 mM of amines and amino acids. As shown in Table S2, several amines with carbon numbers 4 to 8 were accepted as good amino donors. On the contrary, α-amino acids, except for L-Ala, were inert as amino donors. As GABA, ω-amino acids with carbon numbers more than 4 were also accepted as amino donor although those with even-numbered carbons were more active than those with odd-numbered ones. β-Ala aminotransferase from *Bacillus cersus* has been reported to accept even-numbered amino acids, GABA, 6-aminohexanoic acid, and β-Ala, but not 5-aminopentanoic acid [23]. Because our enzyme never uses β-Ala and appears similar to GABA-aminotransferase from its primary structure, VAMT is different from it. No activity was observed for GABA isomers with α- and β-amino group (2-aminobutyric acid, and 3-aminobutyric acid, respectively). Amino acids with ω-amino group, L-Lys and L-Orn, were inert as amino donors, whereas diamines with carbon chains longer than 6 were active. There was no specificity with regard to the structure of the amino donors, although the enzyme preferred primary amines to secondary amines.

Apparent *K*_m_ and *k*_cat_ values of the enzyme for amino donors and amino acceptors were determined when either amino donor or amino acceptor concentration was fixed (Table 2 and Fig. S9). Although L-Ala concentration in the assay (100 mM) was lower than its *K*_m_ value (160 mM), we evaluated kinetic parameters for V and VG by using the data measured under the same amino donor concentration with GABA. The catalytic efficiency (*k*_cat_/*K*_m_) of the enzyme was similar for both VA (pyruvate as an amino acceptor) and V (L-Ala as an amino donor), suggesting that the enzyme catalyzes the reversible amination-deamination reaction between V and VA in accordance with the calculated *E*_a_ values for both reactions. The *K*_m_ value for V increased four times when GABA but not L-Ala was used as an amino donor, but *k*_cat_ value was doubled, resulting in the comparable *k*_cat_/*K*_m_ values. This can be due to the spontaneous conversion of SSA, thus formed by the loss of the amino group of GABA, to cyclic 4-hydroxybutyrolactone and it is not converted back to SSA in the reaction mixture, leading to shift in the reaction equilibrium toward VA formation. Recently, the O'Reilly group used a similar theory to develop a new generation of “smart” amino donors with the ability to form dimer or cyclized forms by transamination, enabling the shift of the reaction equilibrium toward product formation [24]. GABA also appears to be a more suitable amino donor than L-Ala based on the finding that the affinity of GABA to the enzyme was four-times higher than that of L-Ala. The apparent *K*_m_ value for V was 0.48 mM when GABA was used as an amino donor, suggesting that the enzyme has evolved to accept V from the general plant GABA-aminotransferase, which accepts various amino acceptors with sub-millimolar orders of *K*_m_ values [[13], [14], [15]]. Recently, Nakaniwa et al. determined the kinetic parameters of the recombinant VAMT using the same *vamt* (codon non-optimized) as used in this study [20]. The apparent *K*_m_ and *k*_cat_ values for VA formation from V (GABA-dependent) were 0.019 mM and 0.0376 s^−1^, respectively; which were 25.3- and 37.2-folds lower, respectively, than those in our study. However, the calculated *k*_cat_/*K*_m_ value was 1.98 s^−1^/mM, which is comparable with that obtained in this study. Because the assay conditions used to measure the enzyme activity and protein concentrations were very different from each other, we believe that it is not important to compare these values.

**Table 2:** Kinetic parameters of rVAMT.

Varying	Fixed	*K*_m_	*V*_max_	*k*_cat_	*k*_cat_/*K*_m_
substrate	substrate	(mM)	(U/mg protein)	(s^−1^)	(s^−1^/mM)
VA	Na pyruvate^a^	0.25 ± 0.037	2.9 ± 0.11	2.5	9.8
V	GABA^a^	0.48 ± 0.049	1.7 ± 0.051	1.4	3.0
V	L-Ala^a^	0.12 ± 0.019	0.91 ± 0.040	0.77	6.4
Na pyruvate	VA^b^	5.4 ± 0.34	2.7 ± 0.052	2.3	0.43
GABA	V^b^	36 ± 2.1	1.5 ± 0.023	1.3	0.036
L-Ala	V^b^	160 ± 7.0	1.1 ± 0.020	0.93	0.0058
VAG	Na pyruvate^a^	15 ± 4.8	2.3 ± 0.38	1.9	0.13
VG	GABA^a^	3.3 ± 0.56	0.42 ± 0.029	0.36	0.11
VG	L-Ala^a^	1.5 ± 0.37	0.38 ± 0.029	0.32	0.21

Data the mean ± SEM (SEM: fitting error to the Michaelis-Menten equation as calculated the non-linear fitting program).

a At 100 mM concentration.

b At 5 mM concentration.

We next examined whether the glucoside-type compounds, vanillin glucoside (VG) and vanillylamine glucoside (VAG) can be accepted as substrates for VAMT, because several biosynthetic intermediates of capsaicin are accumulated in their glucoside form in *Capsicum frutescens* during growth [25]. The binding affinity of the glucosides was significantly lower than those of the free substrates in both the amination and deamination reactions (Table 2). The *k*_cat_/*K*_m_ values were also lower for the glucoside substrates than those for the free substrates, suggesting that the enzyme prefers the free substrates to the glucoside forms.

Considering VAMT is an aminotransferase, we analyzed the mechanism of binding of its substrates. The Lineweaver–Burk plots for VA-forming activity and GABA concentration with multiple concentrations of V showed parallel lines (Fig. S10), confirming that this enzyme binds to substrates via the ping-pong mechanism [26], as do general aminotransferases [21].

### 3.5 Role of VAMT in capsaicin biosynthesis

The results of BLASTP search revealed high homology of VAMT with putative aminotransferases in plants, for example, *Capsicum*, *Solanum*, *Lycium*, *Datura*, and *Nicotiana*, which belong to the Solanaceae family. In addition, the enzyme showed considerable homology to the GABA aminotransferases from *M. domestica* (cytosolic: GenBank accession no. AFS28621, sequence identity, 77.5%; and mitochondrial: GenBank accession No. AFS28620; 77.4%) [14], *A. thaliana* (mitochondrial: UniProtKB/Swiss-Prot accession no. Q94CE5; 76.1%), and from *Oryza sativa* (cytosolic: UniProtKB/Swiss-Prot accession no. Q7XN11; 75.6%) [27]. However, most of these have not been functionally characterized, except for *S. lycopersicum* GABA-aminotransferases 2 (cytoplasmic; UniProtKB/Swiss-Prot accession no. Q84P53), 1 (mitochondrial; UniProtKB/Swiss-Prot accession no. Q84P54), and 3 (chloroplastic; UniProtKB/Swiss-Prot accession no. Q84P52), exhibiting identities of 84%, 81%, and 80% with VAMT, respectively [28], and the *M. domestica* [14] GABA-aminotransferases. Because no such investigations were carried out, it is not clear whether these plant enzymes with remarkable homologies to VAMT can accept V as an amino acceptor, as VAMT does. However, it is likely that VAMT evolved from a plant GABA-aminotransferase, enabling the acceptance of hydrophobic substrates such as V as amino acceptors. This is also supported by the fact that VAMT did not accept hydrophilic glyoxylate, whereas GABA-aminotransferases do.

Capsaicinoid biosynthesis occurs in placental epidermal cells, and the compounds are secreted toward the outer cell wall, followed by their final accumulation on the placental surface [9,29]. The involvement of *vamt* in the capsaicinoid pathway was examined using feeding experiments, and it was concluded that VAMT actively participates in the capsaicinoid biosynthesis by regulating phenylpropanoid precursors channeled into the pathway [16,30]. An analysis of the cDNA sequence of *vamt* (GenBank accession no. AF085149) revealed that the full-length polypeptide encoded by the gene has no signal sequence, which suggests that VAMT is cytosolically localized. Our finding that VAMT does not accept glucosylated VG or 10.13039/100014690VAG, which may exist in vacuoles, as a good substrate, supports our contention regarding the localization of VAMT. Putative GABA-aminotransferases from other capsaicin-accumulating *Capsicum* species having 98.3–99.8 % identities with VAMT do not have any signal sequence as noted in the BLASTP analysis. It suggests that these proteins might exist in the cytosol and are implicated in capsaicin biosynthesis, although their localization in these plants and their VA-forming activity have not been examined. In contrast, functionally characterized plant GABA-aminotransferases, having remarkable homologies with VAMT, are localized not only in the cytosol, but also in plastids and mitochondria [14,27,28]. Putative GABA-aminotransferases from non-capsaicin accumulating plants belonging to *Solanum* and *Nicotiana* have 83.1–86.7 % and 81.2–85.8 % identities with VAMT, respectively, and are considered to be localized in the cytosol [15,28]. It is likely that such homologs are not responsible for VA formation.

In tomatoes, which belong to the same family as Capsicum, GABA and L-Glu are the most abundant amino acids in the fruit [31,32]. GABA is biosynthesized from L-Glu by the action of glutamate decarboxylase (EC 4.1.1.15) and accumulates in the cytosol. It is then transported into the mitochondria to be further catabolized by the consecutive action of GABA-aminotransferase and succinic semialdehyde dehydrogenase (SSADH; EC 1.2.1.16), named as the GABA-shunt pathway, to succinic acid, which enters the tricarboxylic acid cycle [33,34]. Cytosolic GABA may also be indirectly derived from polyamine metabolism in peroxisomes and plastids [35,36]. Therefore, a sufficient amount of GABA may be present in the cytosol of capsicum fruit as an amino donor of VAMT to accumulate VA as a key intermediate in the biosynthesis of capsaicin. SSA, which is stoichiometrically formed in the VAMT-catalyzed reaction between GABA and V to form VA, is unstable and tends to be coverted spontaneously to cyclic 4-hydroxybutyrolactone, being inactive as an amino acceptor. In plants, SSA can be converted to γ-hydroxybutyrate (GHB) through SSA reductase (SSR) in the cytosol [33,34], which also leads to a shift in the equilibrium of the VAMT reaction toward VA formation. Based on these facts, it can be proposed that VA is formed by the action of VAMT using GABA as an amino donor in the cytosol of capsicum fruit cells.

Gururaj et al. reported the detection of VA-forming activity in *N. tabacum* cells (Solanacecae family) made to express *vamt* through *Agrobacterium*-mediated transformation [19]. This suggested that the gene was functionally expressed in plant tissues although the assay was carried out using crude extract of cells. In a recent study, the functional expression of VAMT was achieved using a gene with non-optimized codon, and the desired VA-forming activity of the recombinant enzyme was confirmed [20]; however, no information on the enzyme characteristics, such as, amino donor/acceptor specificities, reaction toward synthetic glucoside-type compounds, inhibitors, and detailed kinetic parameters, was provided. The VA-forming activity of the placental extract of *C. chinense* was described in the report, but this was also measured using the crude extract [20]. In order to know the physiological functions of VAMT in capsaicin biosynthesis, detailed biochemical analysis of the purified native VAMT remains to be investigated. Purification of VAMT from *C. chinense* fruit is being attempted in our laboratory: it will provide answers for the missing links between native and recombinant enzymes.

## 4 Conclusion

We successfully expressed, purified, and characterized VAMT from a recombinant *E. coli* strain. The enzyme catalyzed reversible transamination reaction between V and VA, and the VA-forming activity was high when GABA was used as an amino donor. The enzyme had maximum activity at pH 8.0 and 55 °C and was stable up to 60 °C at pH in the 4.5–8.0 range. The enzyme was stable in the presence of various chemicals and metal ions. The enzyme accepts several primary amines with carbon numbers of 4–8 and ω-amino acids with carbon chains longer than 4 as amino donors despite the narrow specificity of the amino acceptor. Given the kinetic properties and the predicted subcellular localization, it is likely that VAMT has evolved from GABA-aminotransferase, and is responsible for VA biosynthesis using GABA as an amino donor in the cytosol of capsicum fruit. The findings in this study will provide insights into the activity of VAMT, which should aid the further elucidation of the biosynthetic pathway for capsaicin.

## Funding

This research did not receive any specific grant from funding agencies in the public, commercial, or not-for-profit sectors.

## CRediT authorship contribution statement

**Yasuo Kato:** Writing – review & editing, Writing – original draft, Visualization, Validation, Supervision, Software, Resources, Project administration, Methodology, Investigation, Funding acquisition, Formal analysis, Data curation, Conceptualization. **Taiji Nomura:** Writing – review & editing, Formal analysis, Data curation.

## Declaration of competing interest

The authors declare that they have no known competing financial interests or personal relationships that could have appeared to influence the work reported in this paper.

## Data Availability

Data will be made available on request.
