# Supplementary material for: Functional characterization of Capsicum chinense vanillin aminotransferase: Detection of vanillylamine-forming activity from vanillin
Source: Biochem Biophys Rep. 2024 Mar 25;38:101692. doi: 10.1016/j.bbrep.2024.101692 (PMC10987797; doi:10.1016/j.bbrep.2024.101692)
Supplement: Multimedia component 1 [file mmc1.docx]

**Supplementary Material**

for:

**Functional characterization of *Capsicum chinense* vanillin aminotransferase: Detection of vanillylamine-forming activity from vanillin**

Yasuo KATO* and Taiji NOMURA

Biotechnology Research Center and Department of Biotechnology, Toyama Prefectural

University, 5180 Kurokawa, Imizu, Toyama 939-0398, Japan

*Corresponding author. E-mail address: ykato@pu-toyama.ac.jp

***General procedures***

Silica gel thin-layer (TLC) and column chromatography were performed using TLC 60 F_254_ plates (Merck, Darmstadt, Germany) and silica gel 60 (neutral-type; Kanto Chemical, Tokyo, Japan), respectively. Evaporation and concentration were carried out under reduced pressure at a temperature below 30 °C. ^1^H- and ^13^C-NMR spectra were recorded on an AVANCE 400 spectrometer (Bruker, Billerica, MA, USA) using CDCl_3_, DMSO-d_6_, or D_2_O as a solvent, with tetramethylsilane (TMS) as the internal standard, unless otherwise mentioned. All chemicals were obtained from commercial sources and used without further purification.

***Preparation of vanillylamine glucoside (VAG)***

VAG was chemically synthesized according to the following scheme.

**Figure S1 Chemical synthesis of vanillylamine glucoside (VAG).**

N*-Carbobenzyloxy-vanillylamine*

Vanillylamine hydrochloride (1.0 g, 5.27 mmol) and powdered NaHCO_3_ (2.3 g, 26.4 mmol) were dissolved in 20 mL of H_2_O and to this a CH_2_Cl_2_ solution of carbobenzyloxychloride (2.2 mL, 15.8 mmol) was added at 0 °C with vigorous stirring. The mixture was stirred overnight at room temperature and partitioned using Et_2_O and H_2_O. After extraction with Et_2_O (×3), the combined organic layers were washed with H_2_O, 1 M HCl, H_2_O, and brine, and dried over Na_2_SO_4_. Evaporation and purification using column chromatography (silica, hexane/ethyl acetate = 3/1 → 7/3) gave *N*-carbobenzyloxy-vanillylamine as a colorless oil (1.51 g, 99.8%): ^1^H-NMR (400 MHz, CDCl_3_) *δ* (ppm) 3.85 (s, 3H, CH_3_OAr-), 4.29 (d, 2H, *J* = 5.8 Hz, ArCH_2_NH-), 5.05 (br, 1H, NH), 5.13 (s, 2H, ArCH_2_O-), 5.63 (s, 1H, OH), 6.76 (d, 1H, *J* = 8.1 Hz, Ar), 6.79 (s, 1H, Ar), 6.85 (d, 2H, *J* = 8.0 Hz, Ar), 7.32 (m, 5H, Ar); ^13^C-NMR (100 MHz, CDCl_3_) *δ* (ppm) 45.0 (ArCH_2_NH-), 55.9 (CH_3_OAr-), 66.8 (ArCH_2_O-), 110.5 (Ar), 114.5 (Ar), 120.5 (Ar), 128.2 (Ar), 128.6 (Ar), 130.4 (Ar), 136.6 (Ar), 145.1 (Ar), 146.8 (Ar), 156.5 (-CO-).

*1-*O*-(2-Methoxy-4-carbobenzyloxyaminomethylphenyl)-2,3,4,6-tetra-*O*-acetyl-β-D-glucopyranose*

2 M NaOH (5.5 mL, 11 mmol) was added to a stirred solution of *N*-carbobenzyloxy-vanillylamine (2.8 g, 9.76 mmol) in acetone (30 mL) and the mixture was stirred at room temperature for 10 min. 2,3,4,6-Tetra-*O*-acetyl-α-D-glucopyranosyl bromide (4.1 g, 10 mmol) dissolved in acetone (30 mL) was added, and the mixture was stirred overnight at room temperature. The reaction mixture was evaporated and partitioned using ethyl acetate and H_2_O. The organic layer was washed with H_2_O, 10% citric acid, and brine, and dried (Na_2_SO_4_). Evaporation and purification using column chromatography (silica, hexane/ethyl acetate 3/2 → 1/1)

provided 1-*O*-(2-methoxy-4-carbobenzyloxyaminomethylphenyl)-2,3,4,6-tetra-*O*-acetyl-β-D-glucopyranose as a colorless gum (2.70 g, 44.8%): ^1^H-NMR (400 MHz, CDCl_3_) *δ* (ppm) 2.02 (s, 3H, -O_2_CCH_3_), 2.04 (s, 3H, -O_2_CCH_3_), 2.07 (s, 3H, -O_2_CCH_3_), 2.09 (s, 3H, -O_2_CCH_3_), 3.73 (m, 1H, H-5), 3.75 (s, 3H, CH_3_OAr-), 4.12 (m, 1H, H-6), 4.23 (m, 1H, H-6), 4.32 (d, 2H, *J* = 5.9 Hz, ArCH_2_NH-), 4.89 (m, 1H, H-4), 5.09 (m, 1H, H-2), 5.11 (s, 2H, ArCH_2_O-), 5.28 (m, 2H, H-1, 3), 5.46 (t, 1H, *J* = 9.8 Hz, NH), 6.78 (d, 1H, *J* = 8.0 Hz, Ar), 6.83 (s, 1H, Ar), 7.06 (d, 2H, *J* = 8.1 Hz, Ar), 7.33 (m, 5H, Ar); ^13^C-NMR (100 MHz, CDCl_3_) *δ* (ppm) 20.6 (-O_2_CCH_3_), 20.7 (-O_2_CCH_3_), 20.8 (-O_2_CCH_3_×2), 56.0 (CH_3_OAr-), 61.9 (C-6), 66.9 (ArCH_2_NH-), 68.4 (ArCH_2_O-), 71.0 (C-4), 71.1 (C-2), 72.0 (C-3), 72.3 (C-5), 100.8 (C-1), 112.0 (Ar), 119.6 (Ar), 120.3 (Ar), 128.2 (Ar), 128.6 (Ar), 135.2 (Ar), 145.4 (Ar), 150.8 (Ar), 156.4 (-CO-), 169.4 (-O_2_CCH_3_), 169.7 (-O_2_CCH_3_), 170.2 (-O_2_CCH_3_), 170.7 (-O_2_CCH_3_).

N*-Carbobenzyloxy-vanillylamine glucoside*

NaOMe (50 mg) was added to a solution of 1-*O*-(2-methoxy-4-carbobenzyloxyaminomethylphenyl)-2,3,4,6-tetra-*O*-acetyl-β-D-glucopyranose (7.0 g, 11.3 mmol) in MeOH (100 mL) at room temperature, and the mixture was stirred for 1.5 h. The reaction mixture was acidified to pH 4 by adding Dowex 50 W (H^+^) and then filtered. Evaporation and purification using column chromatography (silica, CHCl_3_/MeOH = 7/3) gave *N*-carbobenzyloxy-vanillylamine glucoside as a colorless powder (1.70 g, 33.5%); ^1^H-NMR (400 MHz, DMSO-d_6_) *δ* (ppm) 3.19 (m, 1H, H-5), 3.25 (m, 3H, H-3, 4, 6), 3.51 (m, 1H, H-6), 3.70 (m, 1H, H-2), 3.75 (s, 3H, CH_3_OAr-), 4.17 (d, 2H, *J* = 4.8 Hz, ArCH_2_NH-), 4.59 (br, 1H, OH), 4.89 (br, 1H, OH), 5.05 (d, 1H, *J* = 5.6 Hz, H-1), 5.08 (s, 2H, ArCH_2_O-), 6.78 (d, 1H, *J* = 8.2 Hz, Ar), 6.91 (s, 1H, Ar), 7.05 (d, 2H, *J* = 8.3 Hz, Ar), 7.36 (m, 5H, Ar), 7.79 (t, 1H, *J* = 4.8 Hz, NH) ; ^13^C-NMR (100 MHz, DMSO-d_6_) *δ* (ppm) 44.0 (ArCH_2_NH-), 56.0 (CH_3_OAr-), 61.1 (C-6), 65.8 (ArCH_2_O-), 70.1 (C-4), 73.6 (C-2), 77.2 (C-3), 77.4 (C-5), 100.6 (C-1), 112.1 (Ar), 115.8 (Ar), 119.6 (Ar), 128.2 (Ar), 128.3 (Ar), 128.8 (Ar), 133.9 (Ar), 137.7 (Ar), 145.8 (Ar), 149.2 (Ar), 156.8 (-CO-).

*Vanillylamine glucoside (VAG)*

Pd/C (100 mg) was added to *N*-carbobenzyloxy-vanillylamine glucoside (2.2 g, 4.89 mmol) dissolved in MeOH (100 mL) under an N_2_ atmosphere and hydrogenated overnight at room temperature. After the removal of the catalysts by filtration, the mixture was evaporated and partitioned with CHCl_3_ and H_2_O. An aqueous phase was washed with CHCl_3_ and lyophilized to obtain VAG as a pale brown powder (1.41 g, 74.1%); ^1^H-NMR (400 MHz, D_2_O) *δ* (ppm: relative to HOD set to 4.87) 3.60 (m, 1H, H-2), 3.64 (m, 3H, H-3, 4, 5), 3.78 (dd, 1H, *J* = 5.6, 12.5 Hz, H-6), 3.91 (s, 2H, ArCH_2_NH-), 3.93 (s, 3H, CH_3_OAr-), 3.95 (m, 1H, H-6), 5.15 (m, 1H, H-1), 6.99 (dd, 1H, *J* = 2.0, 8.3 Hz, Ar), 7.00 (d, 1H, *J* = 2.0 Hz, Ar), 7.19 (d, 2H, *J* = 8.3 Hz, Ar); ^13^C-NMR (100 MHz, D_2_O) *δ* (ppm) 43.9 (ArCH_2_NH-), 55.7 (CH_3_OAr-), 60.3 (C-6), 69.2 (C-4), 72.8 (C-2), 75.4 (C-3), 76.0 (C-5), 100.4 (C-1), 112.3 (Ar), 116.1 (Ar), 120.4 (Ar), 136.0 (Ar), 144.5 (Ar), 148.7 (Ar).

**
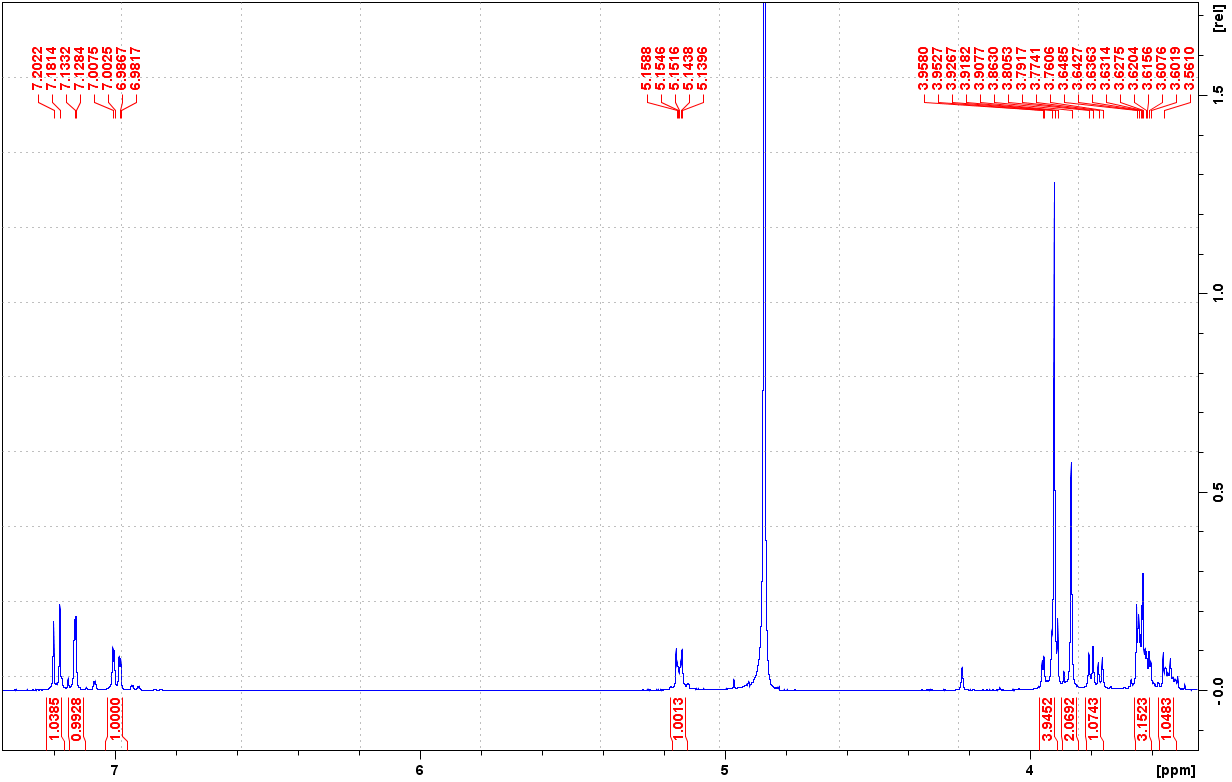
a**

**b**


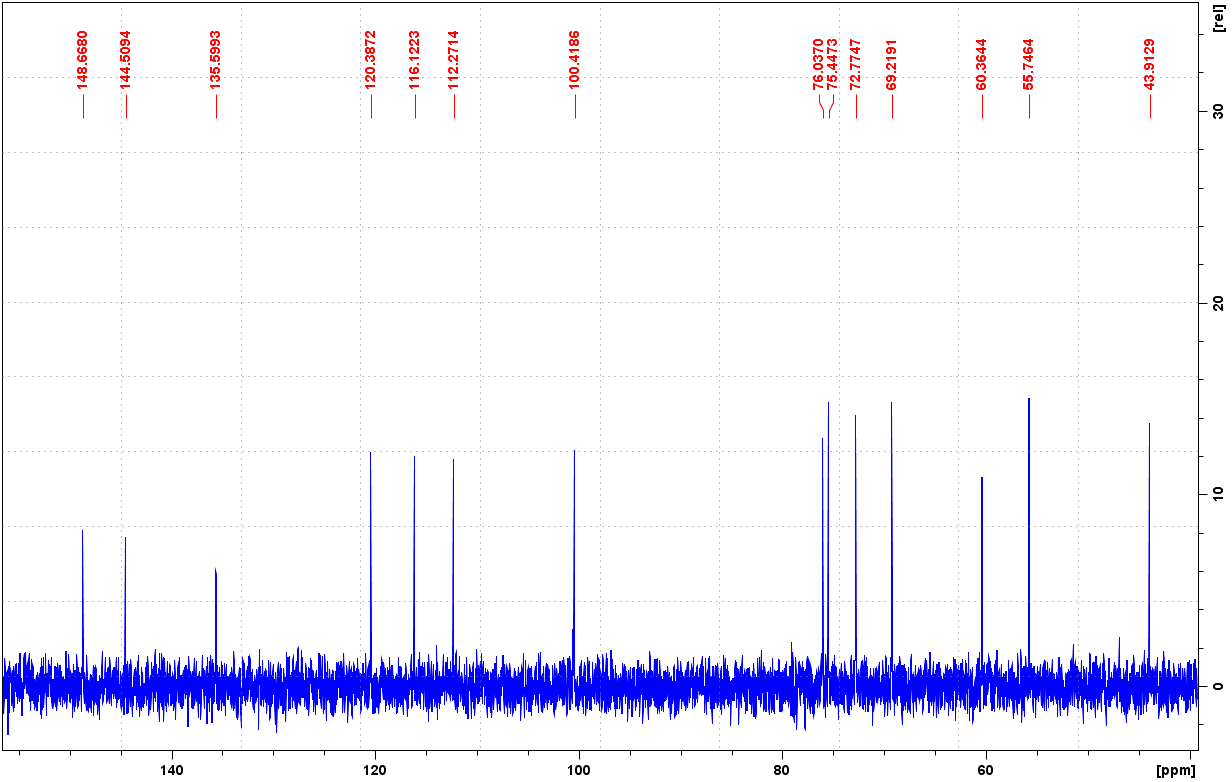


**Figure S2 a) ^1^H- and b) ^13^C-NMR spectra of VAG.**

**Figure S3 HPLC chromatograms of a) V, b) VA, c) VG, and d) VAG.**

**Figure S4 Elution profile of VAMT (▲) on a Superdex 200 Increase column.** Protein standards (●), in the order thyroglobulin (669 kDa), ferritin (440 kDa), aldolase (158 kDa), conalbumin (75 kDa), and ovalbumin (44 kDa).

**Figure S5 Absorption spectra of the purified VAMT.** The absorption spectra of the enzyme were recorded using a Hitachi U-2000 spectrometer with a) the purified recombinant enzyme solution (0.48 mg/mL) in 100 mM KPB (pH 7.5) containing 10 mM 2-ME and 10 μM PLP and b) 100 mM GABA.

**Figure S6 Effects of pH and temperature on the activity (a, c) and stability (b, d) of VAMT for V-forming activity from VA.** The optimum pH and temperature for the enzyme reaction were determined by measuring the enzyme activity for 30 min in 100 mM GTA buffer at pH 3.5–10.0 (every 0.5 pH unit) and at 15–60 °C (every 5 °C) in 100 mM KPB (pH 8.0), respectively. The pH and temperature stabilities of the enzyme were determined by incubating the enzyme for 30 min in 100 mM GTA buffer at pH 3.5–10.0 (every 0.5 pH unit) and for 30 min (●) and 4 h (▲) at 15–60 °C (every 5 °C) in 100 mM KPB (pH 8.0), respectively. The relative activity is expressed as a percentage of the maximum V-forming activity attained under the conditions and the remaining activity is expressed relative to that of the untreated enzyme in the V-forming reaction.

**Figure S7 Effects of pH and temperature on the activity (a, c) and stability (b, d) of VAMT for VA-forming activity from V.** The optimum pH and temperature for the enzyme reaction were determined by measuring the enzyme activity for 30 min in 100 mM GTA buffer at pH 3.5–10.0 (every 0.5 pH unit) and at 15–60 °C (every 5 °C) in 100 mM KPB (pH 8.0), respectively. The pH and temperature stabilities of the enzyme were determined by incubating the enzyme for 30 min in 100 mM GTA buffer at pH 3.5–10.0 (every 0.5 pH unit) and for 30 min (●) and 4 h (▲) at 15–60 °C (every 5 °C) in 100 mM KPB (pH 8.0), respectively. The relative activity is expressed as a percentage of the maximum VA-forming activity attained under the conditions and the remaining activity is expressed relative to that of the untreated enzyme in the VA-forming reaction.

**Figure S8 Arrhenius plots for the calculation of the activation energy (*E*_a_) of VAMT.** a) V-forming reaction with pyruvate as amino donor and VA-forming reactions with b) GABA and c) L-Ala as amino donors, respectively.

**Figure S9 Michaelis–Menten plots of the aminotransferase reactions catalyzed by VAMT****.** a) Dependence of V formation on the concentration of VA in the presence of 100 mM pyruvate. b) Dependence of VA formation on the concentration of V in the presence of 100 mM GABA. c) Dependence of VA formation on the concentration of V in the presence of 100 mM L-Ala. d) Dependence of VG formation on the concentration of VAG in the presence of 100 mM pyruvate. e) Dependence of VAG formation on the concentration of VG in the presence of 100 mM GABA. f) Dependence of VAG formation on the concentration of VG in the presence of 100 mM L-Ala. g) Dependence of V formation on the concentration of pyruvate in the presence of 5 mM VA. h) Dependence of VA formation on the concentration of GABA in the presence of 5 mM V. i) Dependence of VA formation on the concentration of L-Ala in the presence of 5 mM V.Enzyme activity was calculated based on the formation of the reaction product. Correlation coefficient (*R^2^*) in the nonlinear regressions in a), b), c), d), e), f), g), h), and i) was 0.985, 0.994, 0.987, 0.973, 0.990, 0.973, 0.998, 0.998, and 0.999, respectively. See Table 2 for the kinetic parameters, as calculated using nonlinear regression of data to the Michaelis–Menten equation.

**Figure S10 Double reciprocal plots of the enzyme activity against GABA concentration at a series of fixed concentrations of V.** The VA-forming activity was measured with varying GABA concentrations at (●) 0.1 mM, (■) 0.25 mM, (▲) 0.5 mM, (◆) 1 mM, and (×) 2.5 mM of V.

**Table S1 Effect of various compounds on VAMT activity.** The V-forming activity was measured as described in Materials and Methods in the presence of 2 mM of various concentrations of several compounds.

Compound^a^ Concentration Relative activity

(mM) (%)

None - 100

*p*-chloromercuribenzoic acid 2 80

NH_2_OH･HCl 2 63

NiCl_2_ 2 68

CuSO_4_ 2 5.4

1.5 12

1.25 23

HgCl_2_ 2 35

^a^ The enzyme activity was not inhibited (relative activity >85%) by SH-reagents, such as 5,5'-dithiobis(2-nitrobenzoic acid), iodoacetic acid, iodoacetamide, and *N*-ethylmaleimide; chelating reagents, such as *o*-phenanthroline, bipyridyl, 8-hydroxyquinoline, EDTA, *O*,*O*'-bis(2-aminoethyl)ethyleneglycol- *N,N,N*',*N*'-tetraacetic acid (EGTA), and, 1,2-dihydroxy-3,5-benzenedisulfonic acid (Tiron); serine-reagent phenylmethylsulfonyl fluoride; carbonyl reagents, such as NaF, KCN, NaN_3_, hydrazine, and D-cycloserine; reducing reagents, such as DL-penicillamine and dithiothreitol; and metal salts, such as LiCl, H_3_BO_3_, NaCl, MgSO_4_, AlCl_3_, KCl, CaCl_2_, VCl_3_, CrCl_3_, MnSO_4_, FeSO_4_, FeCl_3_, K_4_[Fe(CN)_6_], K_3_[Fe(CN)_6_], CoCl_2_, ZnSO_4_, As_2_O_3_, RbCl, Na_2_MoO_4_, AgNO_3_, CdCl_2_, SnCl_2_, CsCl, Tl_2_SO_4_, BaCl_2_ and PbCl_2_.

**Table S2 Accepted amino donors for VAMT.** The VA-forming activity from V (5 mM) was measured as described in Materials and Methods using 100 mM amino donor. The enzyme activity with GABA was set to 100%.

Amino donor^a^ Relative activity (%)

*n*-butylamine 25

*iso*-butylamine 35

*sec*-butylamine 52

*n*-pentylamine 34

*iso*-pentylamine 39

*neo*-pentylamine 50

*n*-hexylamine 43

cyclohexylamine 31

1,6-diaminohexane 26

*n*-heptylamine 26

1,7-diaminoheptane 33

1,8-diaminooctane 19

------------------------------------------------------------------------

L-Ala 43

GABA 100

5-aminopentanoic acid 17

6-aminohexanoic acid 100

^a^ The following compounds were inert as amino donors at 100 mM concentrations (unless otherwise stated in the parentheses): amines, such as methylamine, ethylamine, ethylenediamine, 2-aminoethanethiol, 2-aminoethanol, taurine, *n*-propylamine, *iso*-propylamine, 1,3-diaminopropane, *tert*-butylamine, 1,4-diaminobutane, 3-aminopentane, 1,5-diaminopentane, agmatine, spermidine, *n*-octylamine, spermine, cinnamoylputrescine, and *p*-coumaroylputrescine, and amino acids, such as D-Ala, L-Asn (16 mM), L-Asp, L-Arg, L-Cys, L-Gln, L-Glu, Gly, L-His, L-Ile (31 mM), L-Leu (18 mM), L-*n*-Leu (18 mM), L-*t*-Leu, L-Lys, L-Met, L-Phe (17 mM), L-Pro, L-Ser, L-Thr (76 mM), L-Trp (5.2 mM), L-Tyr (0.21 mM), L-Val (49 mM), L-*n*-Val (49 mM), L-Orn, β-Ala, 2-aminobutyric acid, and 3-aminobutyric acid.
